# SLC12A5 promotes hepatocellular carcinoma growth and ferroptosis resistance by inducing ER stress and cystine transport changes

**DOI:** 10.1002/cam4.5605

**Published:** 2023-01-16

**Authors:** Qing Tong, Wei Qin, Zheng‐Hao Li, Chun Liu, Zi‐Cheng Wang, Yuan Chu, Xun‐Di Xu

**Affiliations:** ^1^ Hunan Provincial Key Laboratory of Hepatobiliary Disease Research & Division of Hepato‐Biliary‐Pancreatic Surgery, Department of Surgery The Second Xiangya Hospital of Central South University Changsha China; ^2^ Department of Hepato‐Biliary‐Pancreatic Surgery The 3rd Affiliated Teaching Hospital of Xinjiang Medical University (Affiliated Cancer Hospital) Urumqi China; ^3^ Department of General Surgery The South China Hospital of Shenzhen University Shenzhen China

**Keywords:** cystine transport, endoplasmic reticulum stress, ferroptosis, hepatocellular carcinoma, SLC12A5

## Abstract

**Background:**

Hepatocellular carcinoma (HCC) has a poor prognosis and new effective treatments are needed. SLC12A5 plays important roles in multiple complex pathological states and is overexpressed in a variety of malignancies. However, the effects of SLC12A5 in HCC have not been determined.

**Methods:**

SLC12A5 expression was assessed by immunostaining and western blotting. A cell viability assay was used to detect cell proliferation. Flow cytometry was used to evaluate the intracellular calcium concentration and cell cycle. Ferroptosis was detected by transmission electron microscopy, lipid peroxidation, and glutathione assays. Subcutaneous tumor formation experiments were used to validate the tumorigenic effect of SLC12A5 in vivo. RNA‐seq was used to evaluate the molecular mechanisms underlying the effects of SLC12A5. The therapeutic efficacy of targeting *SLC12A5* was assessed in a patient‐derived xenograft (PDX) model.

**Results:**

High SLC12A5 expression was strongly associated with a poor clinical prognosis and promoted HCC growth. Mechanistically, SLC12A5 promoted ER stress to enhance calcium release and upregulated PNCK expression levels. Concomitantly, PNCK was significantly activated by calcium ions released from the ER. PNCK activated and induced the phosphorylation of PI3K/AKT/mTOR pathway components. Furthermore, SLC12A5 inhibited ferroptosis in HCC by upregulating the expression of xCT, a cystine transporter.

**Conclusion:**

High SLC12A5 levels were correlated with a poor prognosis, promoted tumorigenesis, and inhibited ferroptosis in HCC. These findings suggested that SLC12A5 is a therapeutic target and provide insight into the link between ER stress and ferroptosis in HCC.

## Graphical abstract

Ion homeostasis plays a critical role in liver damage and the initiation and progression of hepatocellular carcinoma (HCC). We showed that SLC12A5 (K+‐Cl‐ cotransporter) is overexpressed in HCC and predicts poor prognosis. SLC12A5 promoted ER stress and upregulated PNCK expression levels. PNCK induced phosphorylation of the PI3K/AKT/mTOR pathway components. Furthermore, SLC12A5 inhibited ferroptosis in HCC by upregulating the expression of xCT. SLC12A5 antagonist VU0240551 has translational potential for HCC treatment.

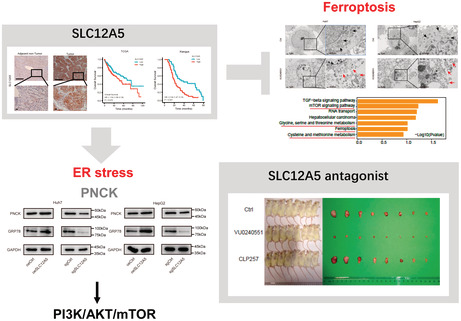

## 1 INTRODUCTION

Hepatocellular carcinoma (HCC) is a gastrointestinal malignancy with a high incidence rate and is responsible for more than 830,000 cancer related deaths each year.^1^, ^2^, ^3^ Thus, the discovery of novel strategies and drug targets for HCC is urgent.^4^, ^5^ Cancer cells exhibit metabolic alterations related to cell growth, proliferation, and death.^6^, ^7^, ^8^ These changes often alter the relative ion homeostasis in HCC cells and regulate the function of ion‐dependent cytokines involved in various biological processes, such as tumor progression, endoplasmic reticulum (ER) stress, and ferroptosis.^9^, ^10^, ^11^

The ER is an essential organelle that serves as the primary site for protein synthesis, cellular development, drug detoxification, and calcium storage.^12^, ^13^ Protein processing, modification, and folding in the ER tightly regulate cell function, fate, and survival.^14^, ^15^ Various physiological and pathological conditions produce alterations in the ER, defined as ER stress.^16^, ^17^, ^18^ ER stress regulates diverse oncogenic, transcriptional, epigenetic, and metabolic processes that together generate pathological microenvironments in stromal and malignant cells.^13^, ^19^ First, an abnormal ion imbalance occurs, followed by ER stress, excessive reactive oxygen species (ROS) generation, oxidative stress injury, and mitochondrial dysfunction.^10^, ^20^ Abnormally activated ER stress could initiate downstream signaling pathways involved in the regulation of tumorigenesis, progression, metastasis, and responses to targeted therapies.^14^

Ferroptosis, a type of regulated cell death, is caused by iron‐dependent excess in lipid peroxides.^21^, ^22^, ^23^ Ferroptosis could occur by two main molecular mechanisms: an intrinsic enzyme‐dependent pathway and extrinsic transporter‐regulated pathway.^24^, ^25^ The intrinsic pathway is initiated mainly by blocking GPX4, an intracellular antioxidant enzyme.^25^ The extrinsic pathway is activated by the suppression of cellular transporters, such as the cystine transporter xCT (also known as SLC7A11), or by the excitation of the iron transporters lactotransferrin and serotransferrin.^25^ Ferroptosis‐sensitive tumor cells have promising therapeutic implications.^26^

SLC12A5, also known as the K^+^‐Cl^−^ co‐transporter KCC2, facilitates the intracellular transport of a broad variety of potassium‐chloride ions.^27^ Previous investigations have demonstrated that the expression of SLC12A5 is elevated in multiple tumor types and its elevated expression is usually correlated with a dismal prognosis.^28^, ^29^ Additionally, SLC12A5 possesses potent oncogenic properties, for example, it promotes tumor growth and metastasis.^30^ Herein, we present a novel molecular mechanism underlying HCC and describe SLC12A5‐mediated crosstalk between ferroptosis and ER stress.

## 2 MATERIALS AND METHODS

### 2.1 Human tissues

Human tissue samples were obtained from surgical resections performed at the Second Xiangya Hospital. The study was approved by the Ethics Committee of the Second Xiangya Hospital (No. LYF2022070).

### 2.2 Animals

Animal experiments were approved by the Animal Care Ethics Committee of the Second Xiangya Hospital (No.2022647). Nude mice (BALB/c, male, 6 weeks old) and NOD‐SCID mice (male, 6 weeks old) were housed under standard conditions with free access to water and feed.

### 2.3 Cell culture

HL7702, PLC/PRF/5, HepG2, Huh7, MHCC‐97H, and HCCLM3 cell lines were purchased from the Cell Bank of the Chinese Academy of Sciences. All cells were grown in cell culture incubators (5% CO_2_ and 37°C). Cells were culture in DMEM (Gibco) containing 10% fetal bovine serum (Biological Industries, Beit‐Haemek).

### 2.4 Hematoxylin–eosin (H&E) staining and Immunohistochemistry (IHC)

H&E staining was performed according to standard methods. The expression of the target protein was assessed by IHC using a PV‐9001 Kit (ZSGB‐BIO). The IHC score was calculated as reported previously.^31^

### 2.5 CRISPR‐Cas9 assay

Lentiviruses carrying a single‐guide RNA (sgRNA) targeting SLC12A5 were purchased from GeneChem. Huh7 and HepG2 cell lines with SLC12A5 knockdown or overexpression were generated by lentivirus infection according to the manufacturer's instructions. Two days after infection, puromycin selection (2 μg/mL) was performed for 1 week. The sgRNA sequences of SLC12A5 are listed in Table S1.

### 2.6 RNAi and gene transfection

Gene‐specific and negative control siRNAs were purchased from RiboBio. Transfection was performed using Lipofectamine 3000 Reagent (Invitrogen). The siRNA sequences are listed in Table S2.

### 2.7 Western blot analysis

Western blotting was performed following the standard protocol. The antibodies used in this study are shown in Table S3. Chemiluminescence signals were detected using an ECL Plus Kit (Thermo Fisher Scientific).

### 2.8 Quantitative reverse transcription polymerase chain reaction (qRT‐PCR) assay

TRIzol (Invitrogen) was used to isolate total cellular RNA. Reverse transcription was conducted using the RT‐PCR Kit (Accurate). Subsequently, real‐time PCR was performed using the SYBR qPCR Kit (Accurate) and LightCycler 480 (Roche). The primers used are listed in Table S4.

### 2.9 RNAseq and bioinformatics

Huh7 and HepG2 cell lines with SLC12A5 knockout or overexpression were obtained. The NovaSeq 6000 platform (Illumina) was used for RNA sequencing. Heatmaps were generated using the R heatmap package based on differential gene expression. A gene set enrichment analysis (GSEA) and gene oncology (GO) analysis were performed to evaluate functional enrichment. Gene expression data sets from TCGA were generated using the public TCGA Research Network.

### 2.10 Cell viability and 5‐ethynyl‐2′‐deoxyuridine incorporation assays

Cells were counted and plated in 96‐well plates. After 2 h of incubation, the cells were treated with the indicated drugs for 24 h. Cell Counting Kit‐8 (CCK‐8) (Dojindo Laboratories) was used according to the kit manual. Absorbance values were estimated at 450 nm. 5‐ethynyl‐2′‐deoxyuridine (EdU) staining was performed using the EdU Apollo567 Kit (RiboBio). Briefly, proliferating cells were stained with Apollo fluorescent dyes, and all the cell nuclei were stained with DAPI.

### 2.11 Lipid peroxidation assay

A lipid peroxidation assay was performed following previously described protocols.^32^ First, cells were collected and plated in 12‐well plates. Subsequently, cells were incubated with complete medium for 24 h. For the next step, fresh medium with 5 μM BODIPY 581/591 C11 dye (Invitrogen) was added. After culture for 30 min in an incubator (37°C, 5% CO_2_), the cells were collected and re‐suspended. Lipid peroxidation levels were analyzed by flow cytometry.

### 2.12 Glutathione assay

Glutathione (GSH) is a tripeptide consisting of glycine, cysteine, and glutamate and an important antioxidant in the cellular system.^33^ The relative GSH and oxidized glutathione disulfide (GSSG) concentrations in tumor cells were determined using a GSH/GSSG Kit (Beyotime). Concisely, samples were incubated with buffer and probe for 1 h at 25°C. Absorbance (412 nm) was measured spectrophotometrically. A standard curve was drawn, and relative GSH concentrations were quantified.

### 2.13 Detection of the intracellular calcium concentration

Intracellular Ca^2+^ ions were measured with the Fluo‐4 AM probe (Beyotime). Briefly, cells were incubated (30 min, 37°C) with 5 Μm Fluo‐4 AM. Subsequently, the intracellular calcium concentration was measured by flow cytometry.

### 2.14 Xenograft tumor models

CRISPR/Cas9 knockdown or overexpression experiments were performed by transduction with lentiviruses. Huh7 cells with SLC12A5 overexpressed (5 × 10^5^)/knockdown(1 × 10^6^) was injected into the right back, and equal amounts of control cells were seeded on the left. Tumor sizes were measured and recorded every 3 days using calipers.

### 2.15 Patient‐derived xenograft experiments

Patient‐derived xenograft (PDX) experiments were conducted under approved guidelines following standard methods.^34^ After tumor establishment in mice, inhibitors (VU0240551) or agonists (CLP257) of SLC12A5 were used. PDX mice were randomized on day 7 into three groups (*n* = 8/group) and treated with different modalities, (i) DMSO, (ii) VU0240551 (0.1 mg/kg), and (iii) CLP257 (1 mg/kg) via intraperitoneal injection twice a week. Tumor growth rates were monitored with a vernier caliper every 3 days. After 21 days of treatment, the mice were sacrificed and tumor tissues were extracted for western blotting and immunohistochemical analyses.

### 2.16 Statistical analysis

All data are shown as the mean ± standard deviation of results from at least three independent experiments. GraphPrism version 8.0 was used for statistical analysis. Overall survival (OS) was analyzed with the Kaplan–Meier method and log rank tests using SPSS V22.0. For all tests, statistical significance was set at *p* < 0.05.

## 3 RESULTS

### 3.1 SLC12A5 is overexpressed in HCC and associated with a poor prognosis

**FIGURE 1:**
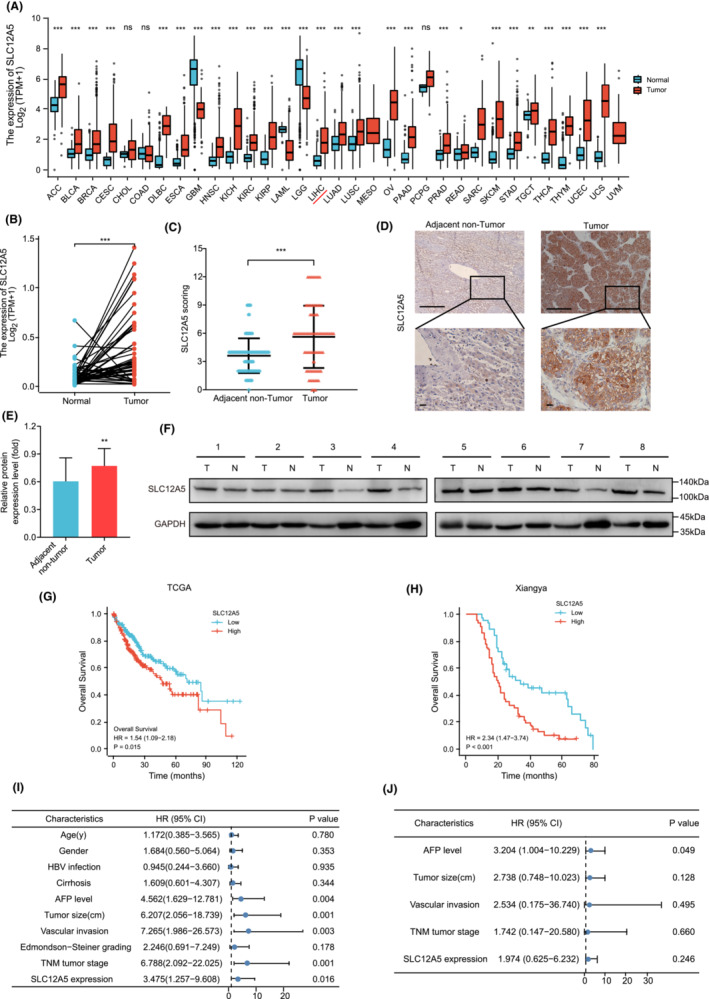
SLC12A5 is overexpressed in HCC and predictive poor clinical prognosis. (A) mRNA levels of the *SLC12A5* of 33 types of tumors in TCGA were analyzed. (B) mRNA expression transcripts per million (TPM) of SLC12A5 in HCC tumor samples (*n* = 371) and the normal adjacent tissues (n = 160) from TCGA. (C–F) Protein levels of SLC12A5 are higher in the paired tumor than in normal adjacent tissue by immunohistochemical staining (C, D) and western blot analysis (E, F). T: Tumors; N: Adjacent non‐tumor. Scale bar, 100 μm. (G, H) Overall survival curves for HCC tissues with different SLC12A5 expression levels in two independent cohorts: Cohort 1 (TCGA cohort, *n* = 373) and Cohort 2 (Xiangya cohort, *n* = 102). (I, J) Univariate and multivariate Cox analyses of independent predictors associated with OS in the Xiangya cohort. Data are presented as mean ± SD. *p < 0.05; **p < 0.01; ***p < 0.001. Mann–Whitney *U* test (A, B). Two‐tailed paired Student's *t*‐tests (C, E). TCGA, The Cancer Genome Atlas; Cancer type abbreviations follow TCGA standards such as Liver Hepatocellular Carcinoma (LIHC); IHC, immunohistochemistry; OS, overall survival; AFP, alpha‐fetoprotein; TNM, tumor‐node‐metastasis; CI, confidence intervals.

**FIGURE 2:**
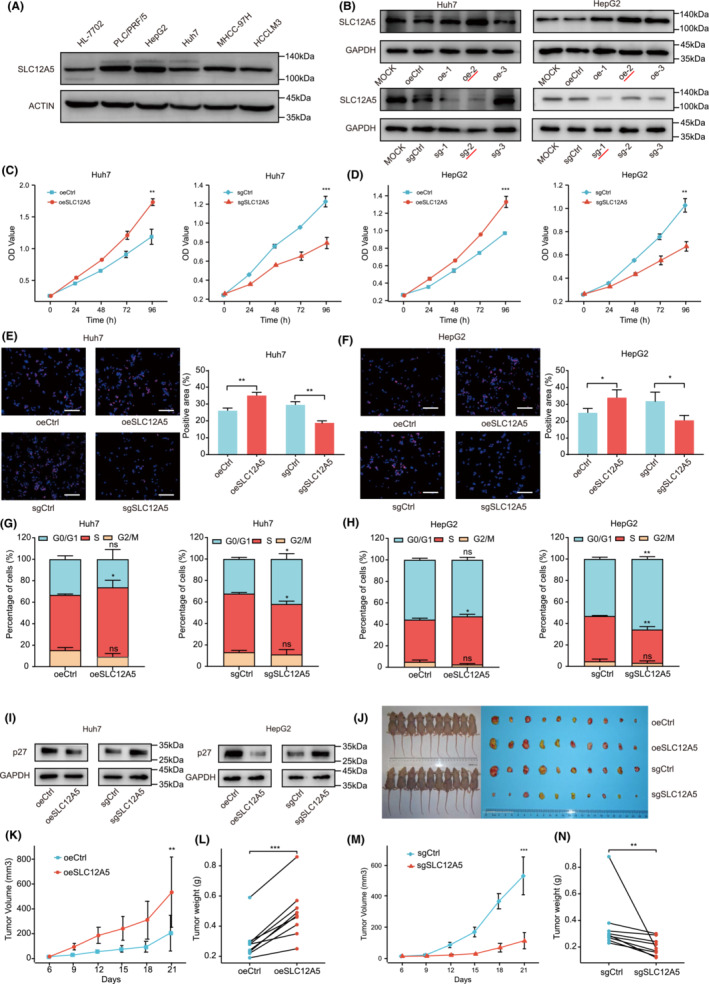
SLC12A5 promotes HCC malignant growth in vitro and in vivo. (A) Western blot analysis of SLC12A5 expression in HL‐7702, PLC/PRF/5, HepG2, Huh7, MHCC‐97H, and HCCLM3 cell lines. (B) Western blot analyses of SLC12A5 protein levels in *SLC12A5* gene overexpression and knockdown cells and control cells. Cells labeled with red lines were used for subsequent experiments. (C, D) Proliferation of SLC12A5 overexpression and knockdown cells was determined by the CCK‐8 assay. (E, F) EdU incorporation assays (scale bar indicates 100 μm) in oeSLC12A5, sgSLC12A5, and control cells. (G, H) Cell cycle status was measured. (I) Cell cycle inhibitory protein p27 levels were detected using western blotting. (J) The tumor formation assay was used to elucidate the function of SLC12A5 in vivo. (K–N) Tumor volume and weight were assessed. Data are represented as mean ± SD. *p < 0.05; **p < 0.01; ***p < 0.001. Two‐way ANOVA (C, D, K, M). Two‐tailed, unpaired (E, F, G, H) or paired (L, N) Student's *t*‐tests.

We evaluated the mRNA levels of *SLC12A5* in 33 primary tumor types using the open‐access dataset of The Cancer Genome Atlas (TCGA). The mRNA level of SLC12A5 was elevated in 24 tumor types compared with levels in normal tissues (Figure 1A). Furthermore, we found that the SLC12A5 mRNA levels were higher in HCC tumor tissues than in paired adjacent non‐tumor tissues (Figure 1B). To further validate the results of bioinformatic analyses, we performed an IHC assay to analyze SLC12A5 expression in 109 patients with HCC (Xiangya cohort) and corresponding adjacent tissues (Table S5). SLC12A5 protein expression levels of the Xiangya cohort were significantly higher than in the control group (*p* < 0.001) (Figure 1C, D). Moreover, the expression of SLC12A5 was higher in 24 HCC tissues than in paired adjacent non‐tumor tissues, as determined by western blotting (*p* < 0.01) (Figure 1E, F and Figure S1). Similarly, in various human HCC cell lines (PLC/PRF5, HepG2, Huh7, MHCC‐97H, and HCCLM3), SLC12A5 protein expression levels were significantly higher than those in a normal human liver cell line (HL‐7702) (Figure 2A and Figure S2A). A Kaplan–Meier survival curve for OS indicated that patients with high SLC12A5 expression levels had a particularly poor prognosis (Figure 1G, H). Subsequently, univariate and multivariate Cox analyses of the Xiangya cohort were performed (Figure 1I, J). The Cox regression analysis also suggested that SLC12A5 was an indicator of a poor prognosis. The prognostic factors identified in the univariate analysis were subjected to a multivariate regression analysis. Taken together, SLC12A5 may act as an oncogenic driver and novel diagnostic biomarker in HCC.

### 3.2 SLC12A5 enhances HCC cell growth and cell cycle progression via the inhibition of p27 expression

Based on SLC12A5 expression levels, Huh7 (low) and HepG2 (high) cells were selected for the following experiments (Figure 2A and Figure S2A). To further determine the function of SLC12A5 in HCC, the CRISPR/Cas9 system was used to establish stable transgenic cell lines with SLC12A5 overexpression or knockdown. The transfection efficiency was confirmed by western blotting (Figure 2B and Figure S2B–E). CCK‐8 and EdU assays revealed that SLC12A5 overexpression promoted growth, whereas SLC12A5 knockdown significantly suppressed proliferation (Figure 2C–F). Subsequently, cell cycle profiles were monitored by flow cytometry. SLC12A5 overexpression induced cell cycle progression, and SLC12A5 knockdown arrested malignant proliferation at the S stage (Figure 2G, H). This finding was verified by western blotting, indicating that SLC12A5 indirectly downregulated p27, a key inhibitor of the cell cycle (Figure 2I and Figure S2F–G).

To examine the role of SLC12A5 in tumor initiation, we next considered the impact of SLC12A5 overexpression or knockdown on tumorigenicity in a nude mouse xenograft model (Figure 2J). The tumor growth rate was faster in the SLC12A5 overexpression group than in the control group, whereas the knockdown of SLC12A5 had the opposite effect (Figure 2K, M). The result for final tumor weights were in line with the results of the tumor growth curve analysis (Figure 2L, N). Collectively, these results demonstrated that SLC12A5 expression was positively correlated with proliferation and cell cycle progression in vitro and in vivo.

### 3.3 VU0240551, a selective SLC12A5 antagonist, enhance ferroptosis activity in HCC

**FIGURE 3:**
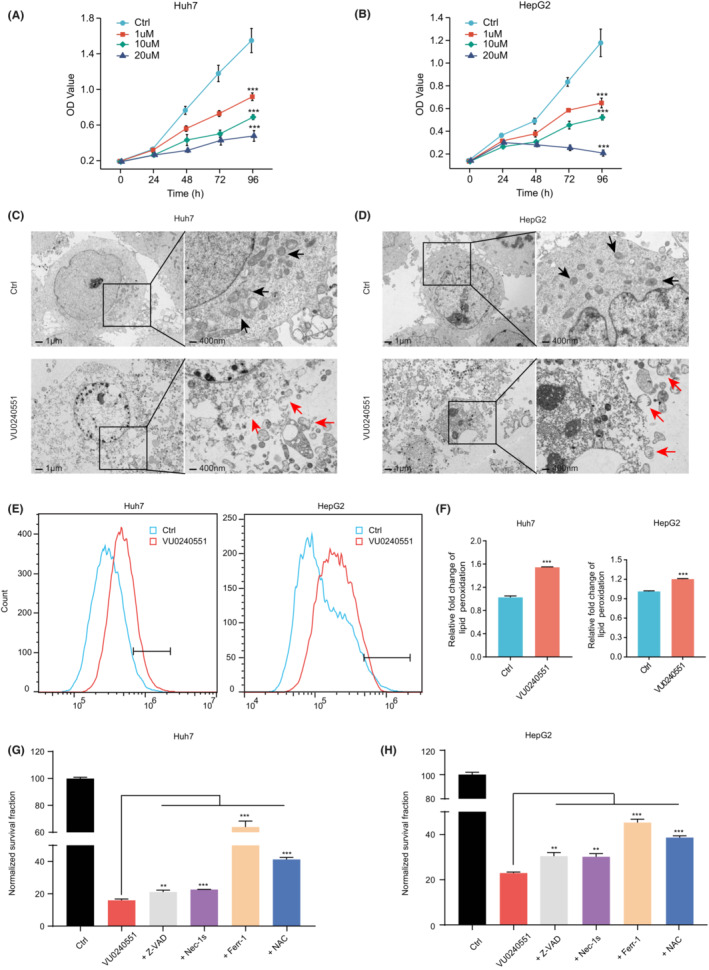
VU0240551 induces ferroptosis in cancer cells. (A, B) Cell viability was assessed by CCK‐8 assays. (C, D) Transmission electron microscope images of the Huh7 and HepG2 at 24 h after incubation with VU0240551. Black arrows, normal mitochondria; red arrows, damaged mitochondria with the reduction or disappearance of mitochondrial cristae. (E, F) After 24 hours of Huh7 and HepG2 cell lines incubation with VU0240551 (1 μM), lipid peroxidation was assessed. Bar histograms show the relative level (F). G, H Cell viability assay in Huh7 and HepG2 that were pretreated with Z‐VAD‐fmk (Z‐VAD, 5 μM), necrostatin‐1 s (Nec‐1 s, 2 μM), ferrostatin‐1 (Ferr‐1, 5 μM), N‐acetyl‐L‐cysteine (NAC, 5 mM), or DMSO for 24 h followed by exposure to VU0240551 (1 μM). The cell viability data were normalized to the control group. Data are presented as mean ± SD. **p < 0.01; ***p < 0.001. Two‐way ANOVA (A, B). Two‐tailed, unpaired Student's *t*‐tests (F, G, H).

Considering that *SLC12A5* is an oncogene, we investigated whether VU0240551, a selective SLC12A5 antagonist, could prevent growth and induce cancer cell death. Cell viability was related to the concentration of VU0240551 (Figure 3A, B). Transmission electron microscopy revealed that Huh7 and HepG2 cells after VU0240551 intervention exhibited damaged and ruptured mitochondria, with intact nuclei, which are morphological features of ferroptosis (Figure 3C, D). Given that the increase in lipid peroxidation is a typical feature of ferroptosis, the lipid ROS level was compared between the drug intervention and control groups in Huh7 and HepG2 cells. Flow cytometry fluorescence intensity plots indicated that the baseline peak range of lipid ROS was increased in Huh7 and HepG2 incubated with VU0240551 (Figure 3E, F). To further analyze the cell death mechanism in VU0240551‐treated cancer cells, the impact of common cell death inhibitors was evaluated. The greatest improvement was noted for ferrostatin‐1 treatment (Figure 3G, H). Therefore, we believe that VU0240551 induces ferroptosis in HCC cells.

### 3.4 SLC12A5 inhibits HCC ferroptosis mainly by regulating the glutathione system

**FIGURE 4:**
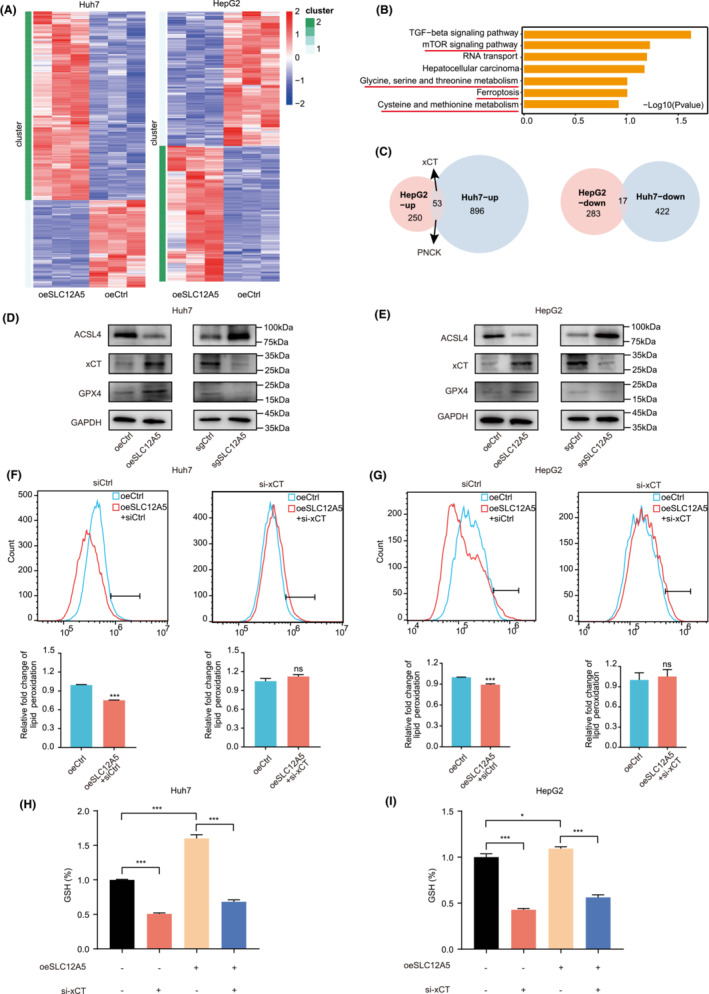
SLC12A5 regulates ferroptosis by promoting xCT to alter cystine transport. (A) Heatmap of transcriptional changes in oeSLC12A5 and control cells. (B) KEGG analysis showing the significantly modulated signaling pathways after SLC12A5 overexpression in Huh7 and HepG2 cells. (C) xCT/PNCK expression was induced by SLC12A5 in HCC by overlapping RNA‐seq data between the Huh7 and HepG2 cell lines. (D, E) Representative western blotting of three ferroptosis proteins. (F, G) Lipid peroxidation assessment in oeCtrl, oeSLC12A5, and oeSLC12A5 + si‐xCT. (H, I) Determination of reduced glutathione (GSH) levels. The presented data are mean ± SD. *p < 0.05; ***p < 0.001. Two‐tailed unpaired Student's *t*‐tests (F, G). One‐way ANOVA (H, I).

Next, we examined the molecular mechanism by which SLC12A5 regulates ferroptosis in HCC cells. We used the RNA‐seq approach to analyze Huh7 and HepG2 cells with SLC12A5 overexpression or knockdown (Figure 4A and Figure S3A, B). Interestingly, RNA‐seq revealed a major impact on transcripts associated with cysteine and glycine metabolism (Figure 4B). Owing to overlap in RNA‐seq results for Huh7 and HepG2 cells, we focused on xCT, a cystine transporter (Figure 4C). The results obtained by RNA‐seq were validated using RT‐qPCR and western blotting (Figure 4D, E and Figure S3C–F). Based on the RNA‐seq data, we hypothesized that SLC12A5 might be involved in ferroptosis by regulating glutathione metabolism. We tested molecular pathways involved in xCT‐induced ferroptosis. SLC12A5 increased the relative expression of GPX4, and reduced the relative expression of ACSL4(Figure 4D, E and Figure S3E–F). We further tested whether SLC12A5 could affect lipid peroxidation by regulating xCT in Huh7 and HepG2 cells (Figure 4F, G). SLC12A5 overexpression upregulated GSH levels, and xCT knockdown reversed the effect of SLC12A5 overexpression on GSH levels (Figure 4H, I). Collectively, the above experiments indicated that SLC12A5 inhibits ferroptosis in HCC mainly by regulating the glutathione system via the xCT transporter.

### 3.5 SLC12A5 promotes HCC growth by inducing ER stress and enhancing pregnancy up‐regulated non‐ubiquitously‐expressed CaM kinase (PNCK) expression

**FIGURE 5:**
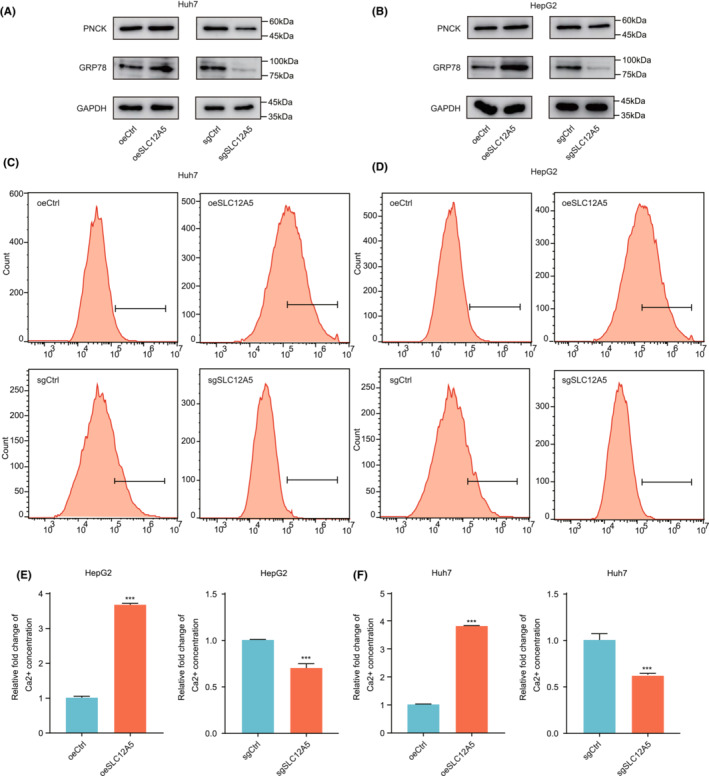
SLC12A5 induces ER stress to release calcium ions and activate PNCK. (A, B) Western blotting validation of ER stress and candidate molecules. (C, D) Intracellular Ca^2+^ concentration was monitored by flow cytometric analysis using Fluo‐4 AM dye. (E, F) Quantitative analysis of intracellular Ca^2+^ concentration. Data presented are mean ± SD. ***p < 0.001. Two‐tailed unpaired Student's *t*‐tests (E, F).

**FIGURE 6:**
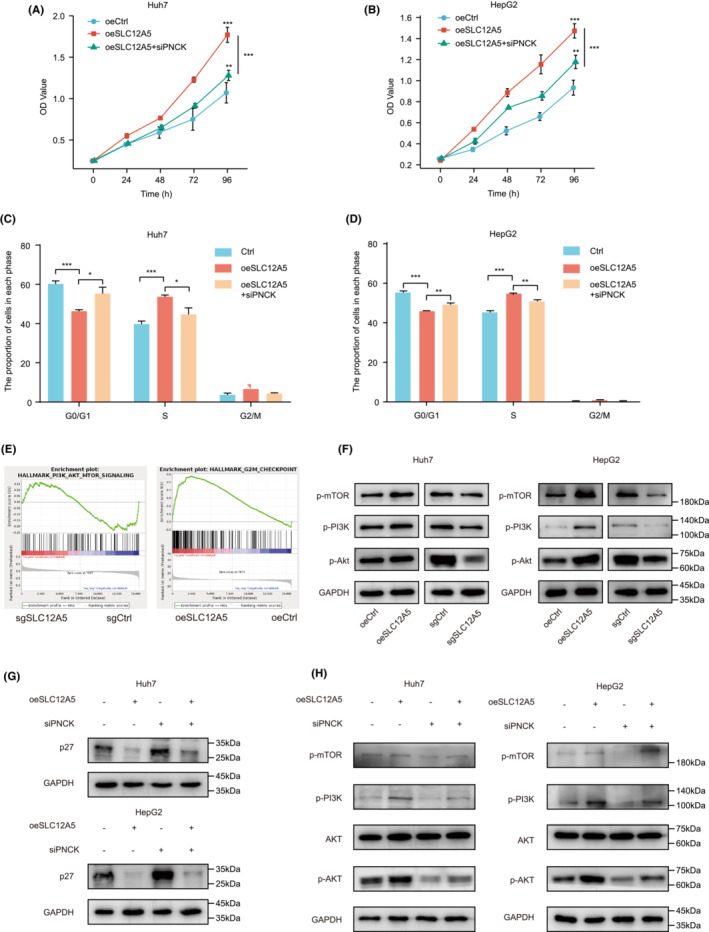
SLC12A5 regulates PNCK to activate PI3K/Akt signaling by inducing ER stress. (A, B) Relative proliferative capacity was measured by CCK‐8 assay. (C, D) Cell cycle distribution was determined by flow cytometry. (E) GSEA for RNA‐seq data (HepG2 cells). (F) Western blotting of the PI3K/Akt signaling pathway. (G) Protein levels of p27 were detected by western blotting. (H) The levels of PI3K/Akt/mTOR signaling pathway components were determined using western blotting. Data are presented as mean ± SD. *p < 0.05; **p < 0.01; ***p < 0.001. Two‐way ANOVA (A, B) and one‐way ANOVA (C, D).

Next, we investigated the mechanism by which SLC12A5 promotes HCC aggressiveness. First, PNCK was identified as a downstream molecule of SLC12A5 based on overlap in mRNA‐seq datasets (Figure 4C). PNCK, a Ca^2+^/calmodulin‐dependent kinase, is activated by Ca^2+^ released by ER stress.^35^ Indeed, we confirmed that the PNCK protein expression level was positively correlated with the SLC12A5 expression level using western blotting. In addition, increased SLC12A5 expression levels led to ER stress (Figure 5A, B and Figure S4A–D). The Ca^2+^ concentration determined using Fluo‐4 AM staining suggested that SLC12A5 promotes ER Ca^2+^ release into the cytosol to activate PNCK (Figure 5C–F). Subsequently, we found that the knockdown of PNCK attenuated the SLC12A5 induced increase in proliferation (Figure 6A, B). Likewise, cell cycle progression was inhibited by PNCK knockdown in SLC12A5 overexpressing‐cancer cells (Figure 6C, D).

### 3.6 SLC12A5 mediates PNCK‐induced phosphorylation of PI3K/Akt/mTOR signaling by triggering ER stress

We performed a GSEA of our RNA‐Seq dataset, focusing specifically on gene sets involved in PI3K/AKT/mTOR signaling and on cell cycle pathway genes (Figure 6E). Indeed, we confirmed, using western blotting, that SLC12A5 can activate the PI3K/AKT/mTOR signaling pathway (Figure 6F and Figure S6A–D). A previous study has shown that PNCK could phosphorylate IκBα and trigger NF‐κB activation.^35^ Therefore, we assumed that PNCK could also activate the PI3K/AKT/mTOR pathway via phosphorylation. Interestingly, our data indicated that PNCK might inhibit p27 and activate PI3K/AKT/mTOR signaling via phosphorylation (Figure 6G, H, and Figure S6E–H). Together, these results showed that SLC12A5 increased *PNCK* gene expression and activation by triggering ER stress and that PNCK could activate and phosphorylate the PI3K/AKT/mTOR pathway.

### 3.7 VU0240551 has translational potential for HCC treatment

**FIGURE 7:**
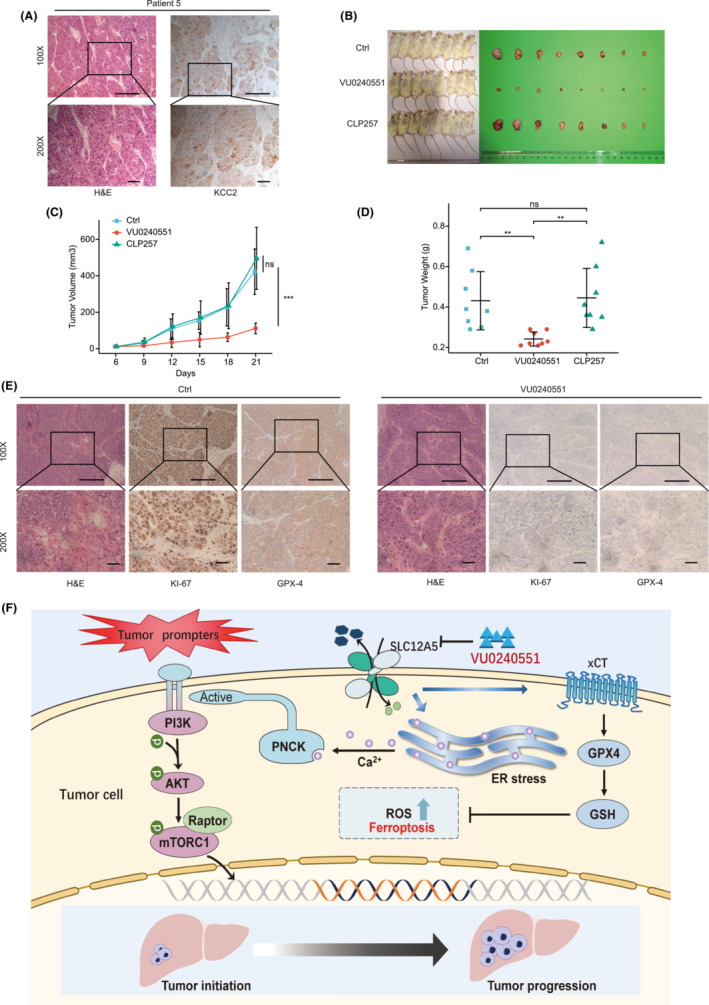
Pharmacological therapies for targeting SLC12A5 in PDX model. (A) H&E and immunohistochemical staining in the PDX models for primary tumor. (B) Photographs of tumors. (C) Tumor volumes were calculated and tumor growth curves were plotted. (D) Tumor weight was measured at the end point. (E) H&E and immunohistochemical staining the histology of tumor tissues. (F) A schematic diagram illustrates the mechanisms to regulate the malignancy and ferroptosis resistance of HCC. Data are presented as mean ± SD. **p < 0.01; ***p < 0.001. Two‐way ANOVA (C) and one‐way ANOVA (D).

Given the significant clinical correlates of SLC12A5, we investigated the potential for clinical translation by evaluating the treatment of HCC with VU0240551. The viability of Huh7 and HepG2 cells decreased with increasing densities of VU0240551 (Figure 3A, B). A cell cycle analysis by flow cytometry confirmed that VU0240551 arrested cells in the S phase (Figure S7A–B). Furthermore, VU0240551 was less toxic to normal liver cells than to cancer cells (Figure S7C). We used a PDX model to validate the efficacy and safety of VU0240551. PDX tumors were derived from a patient with high SLC12A5 expression in tumor tissues (Figure 7A). No toxicity was observed during the study, as mice did not exhibit body weight loss (Figure S7D). In addition, tumor shrinkage was observed in the VU0240551‐treated group (Figure 7B). The tumor growth rate decreased significantly on treatment with VU0240551 targeting SLC12A5 (*p* < 0.001) (Figure 7C). The mice treated with VU0240551 also had lower tumor weights than mice treated with DMSO or CLP257 (Figure 7D). We also evaluated KI‐67 and GPX4 protein expression levels in tumors by IHC, revealing that VU0240551 efficiently prevented tumor growth and induced tumor ferroptosis (Figure 7E). Collectively, these results suggested that targeting SLC12A5 represents a promising avenue for the treatment of HCC, with translational potential.

## 4 DISCUSSION

Our data showed that *SLC12A5* is a novel oncogene that regulates proliferation, cell cycle progression, ER stress, PI3K/AKT/mTOR signaling, and resistance to ferroptosis in HCC. Mechanistically, high SLC12A5 expression disrupted intracellular ion homeostasis, induced intracellular ER stress, and lSed to the upregulation of PNCK expression. Following this, Ca^2+^ was released from the ER into the cytoplasm, consequently activating PNCK. Furthermore, PNCK activated PI3K/Akt/mTOR signaling via a phosphorylation‐dependent mechanism to promote tumorigenesis. Oncogenic roles of SLC12A5 have been reported in the literature. For example, SLC12A5 can interact with and enhance SOX18 activity to promote bladder cancer progression via the NF‐κB/MMP‐7 pathway.^29^, ^36^ PNCK could enhance tumor cell proliferation, which is dependent on PTEN protein phosphatase activity.^37^ Another study has shown that the inhibition of PNCK might delay proliferation and accelerate apoptosis in cancer cells by regulating the PI3K/AKT/mTOR signaling pathway.^38^ Our experimental findings confirmed that the pathway activation was caused by PNCK‐induced phosphorylation. We also identified SLC12A5 as an upstream regulator of PNCK that is activated by Ca^2+^ release during ER stress. Our findings extend previous research and provide new insights into the effects and mechanism of action of SLC12A5.

Ferroptosis is a novel pattern of programmed cell death that depends on iron overload.^23^ Ferroptosis has sparked great interest because it offers new therapeutic targets.^39^ A study has shown that dihydroorotate dehydrogenase attenuates ferroptosis induced by GPX4 inhibition in cancer cells.^21^ Legumain, a conserved asparaginyl endopeptidase, promotes autophagy of GPX4.^40^ Sorafenib might inhibit xCT and trigger ER stress and ferroptosis; however, the underlying mechanism is not well understood.^41^ Here, we reported that SLC12A5 could induce ER stress to promote tumor progression and ferroptosis resistance; alternatively, VU0240551, a SLC12A5 inhibitor, could induce ferroptosis via the down‐regulation of xCT.

The relationship between ROS/ferroptosis and ER stress is very complex. The processes might have different roles at various stages of cancer. The ER stress response in cancer cells is advantageous for minimizing damage.^42^ Recently, various pathogenic processes have been linked to ER stress, including inflammation, immunity, tumorigenesis, apoptosis, and autophagy.^15^, ^43^, ^44^ Nonetheless, the accurate pathophysiology of ER stress in ferroptosis is not yet known. Previous reports have noted that ER stress induces ROS.^45^, ^46^ Elevated lipid ROS levels are a prerequisite for ferroptosis.^47^, ^48^ In contrast, other studies have shown that ROS may trigger an ER stress response.^49^, ^50^ It is generally accepted that ER stress can induce cellular reprogramming to adaptation or death.^14^ Based on previous reports, we hypothesized that ER stress may be correlated with ferroptosis. In this study, we demonstrated that SLC12A5 triggers ER stress to release Ca^2+^, thereby promoting tumor progression by activating PNCK. Moreover, we found that SLC12A5 promotes intracellular GSH synthesis and alleviates lipid peroxidation to inhibit ferroptosis by upregulating xCT transcription. These results further expand our understanding of the multifaceted role of ER stress, which may promote ferroptosis‐resistance in cancer cells. We performed various experiments to investigate the relationship between ER stress and ferroptosis in HCC as well as the mechanism underlying the effect of SLC12A5 in HCC. However, interactions between ferroptosis and ER stress are still far from being completely understood. Further studies are required to resolve this issue.

## AUTHOR CONTRIBUTIONS

**Qing Tong:** Conceptualization (lead); data curation (lead); formal analysis (lead); funding acquisition (supporting); investigation (lead); methodology (lead); project administration (lead); resources (lead); software (lead); supervision (lead); validation (equal); visualization (lead); writing – original draft (lead); writing – review and editing (lead). **wei qin:** Data curation (supporting). **Zhenghao Li:** Software (equal). **Chun Liu:** Validation (supporting). **Zicheng Wang:** Writing – review and editing (supporting). **Yuan Chu:** Data curation (supporting); formal analysis (supporting). **Xundi Xu:** Funding acquisition (lead); project administration (lead); validation (lead); writing – review and editing (equal).

## CONFLICT OF INTEREST

The authors declare that they do not have any conflict of interest.

## ETHICAL APPROVAL

Our study was approved by the Medical Ethics Committee of the Second Xiangya Hospital (No. LYF2022070).

## Supporting information


Figure S1.

Figure S2.

Figure S3.

Figure S4.

Figure S5.

Figure S6.

Figure S7.

Table S1.

Table S2.

Table S3.

Table S4.

Table S5.

## Data Availability

The data for our article is given in this paper and its additional files.
