# Supplementary figures and images for: SLC12A5 promotes hepatocellular carcinoma growth and ferroptosis resistance by inducing ER stress and cystine transport changes
Source: Cancer Med. 2023 Jan 16;12(7):8526–41. doi: 10.1002/cam4.5605 (PMC10134347; doi:10.1002/cam4.5605)

Figure S1

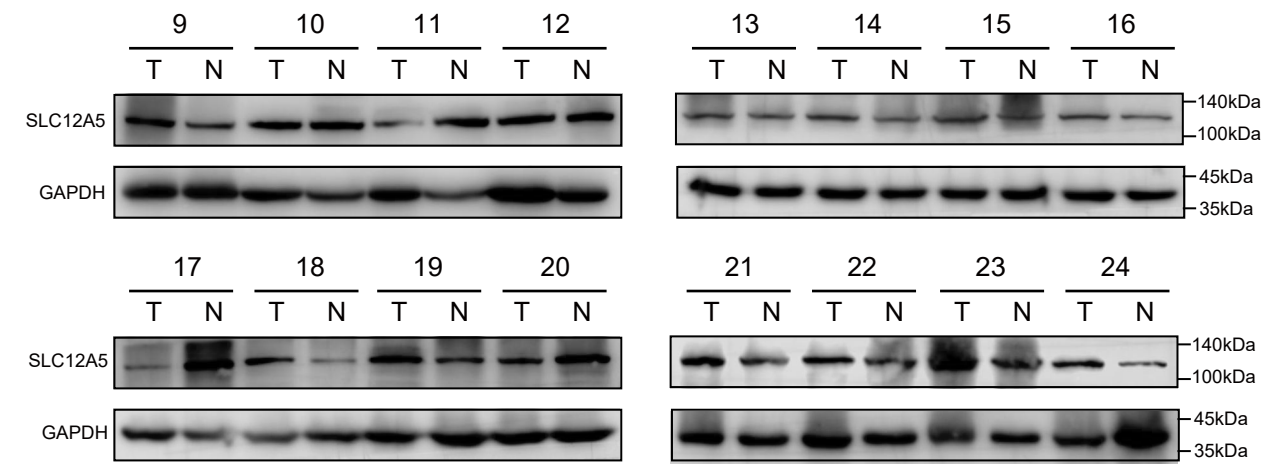

Supplement: Supplementary file 1 — Figure S1. [file CAM4-12-8526-s005.pdf]

Figure S2

A

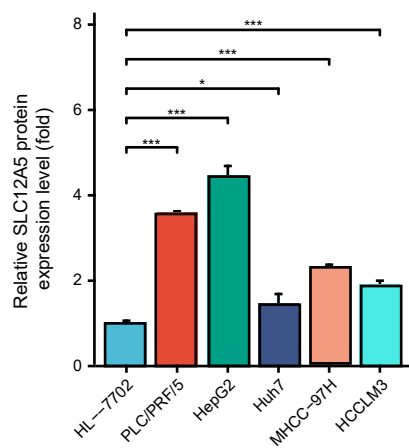

B

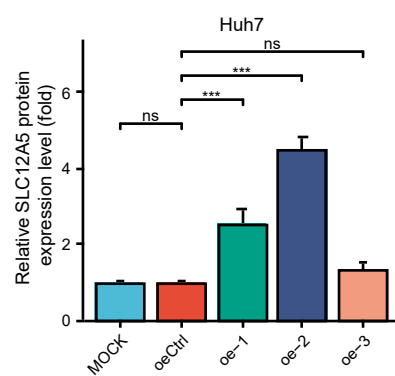

C

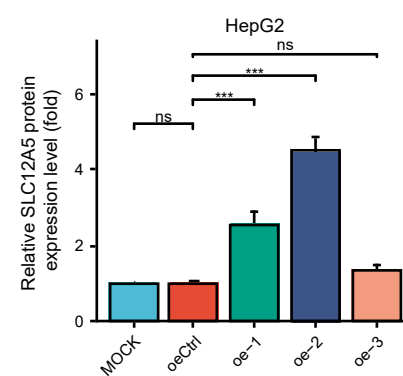

D

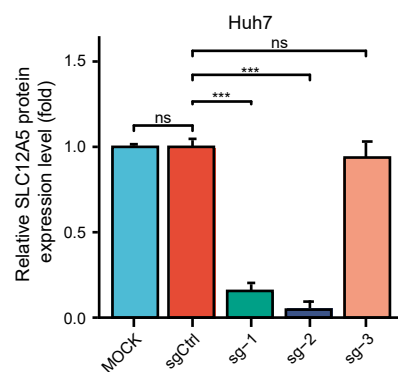

E

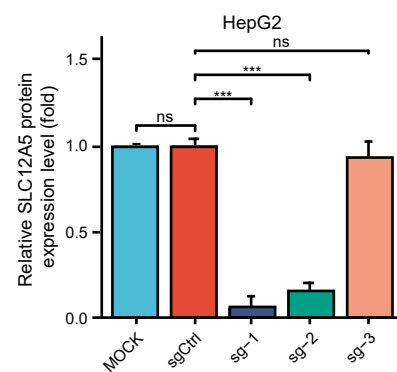

F

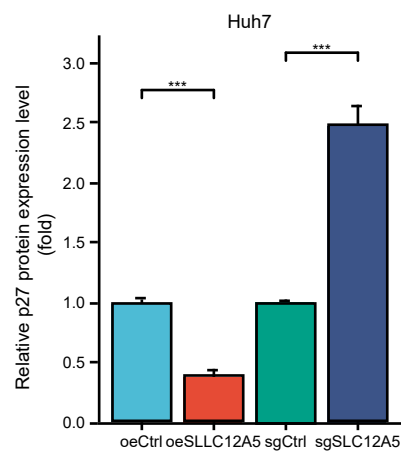

G

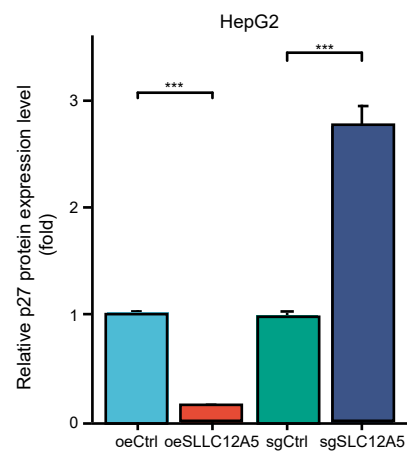

Supplement: Supplementary file 2 — Figure S2. [file CAM4-12-8526-s009.pdf]

Figure S3

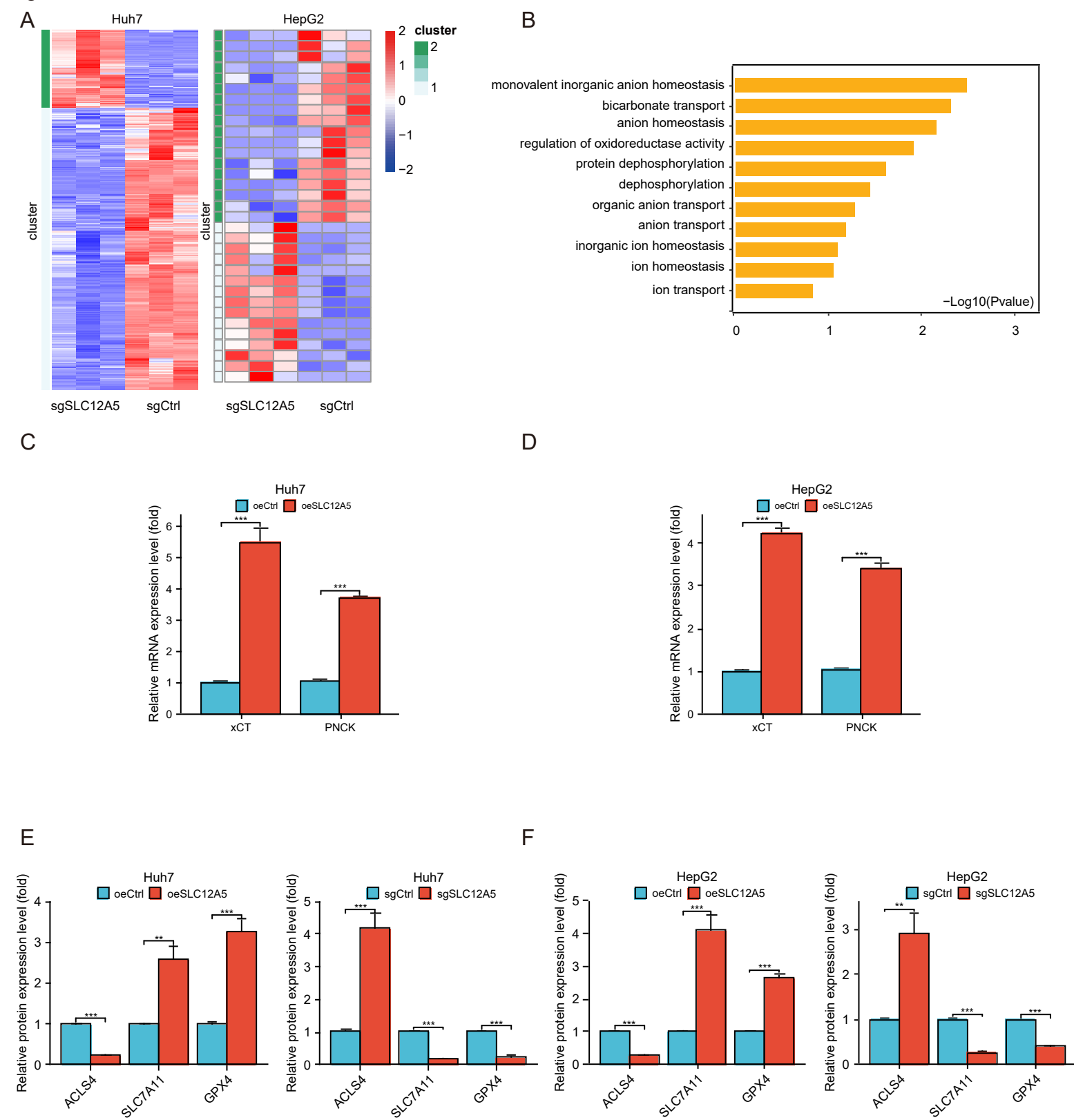

Supplement: Supplementary file 3 — Figure S3. [file CAM4-12-8526-s012.pdf]

Figure S4

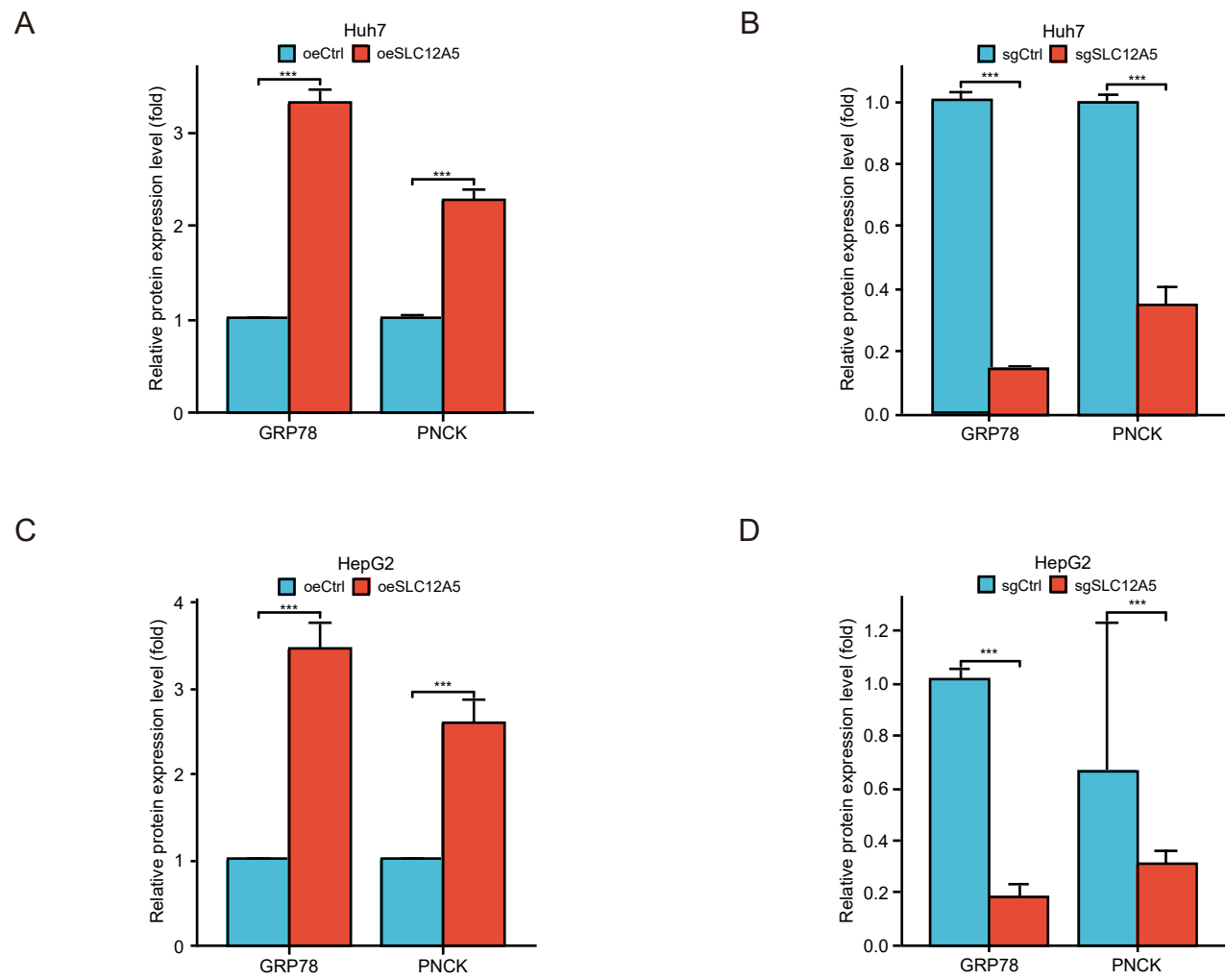

Supplement: Supplementary file 4 — Figure S4. [file CAM4-12-8526-s007.pdf]

Figure S5

A

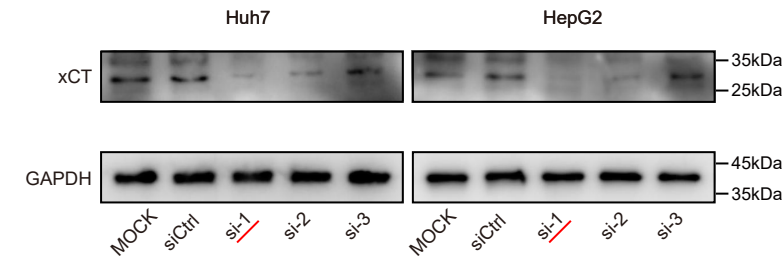

B

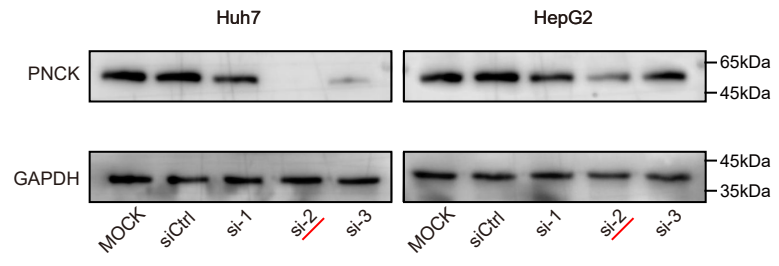

C

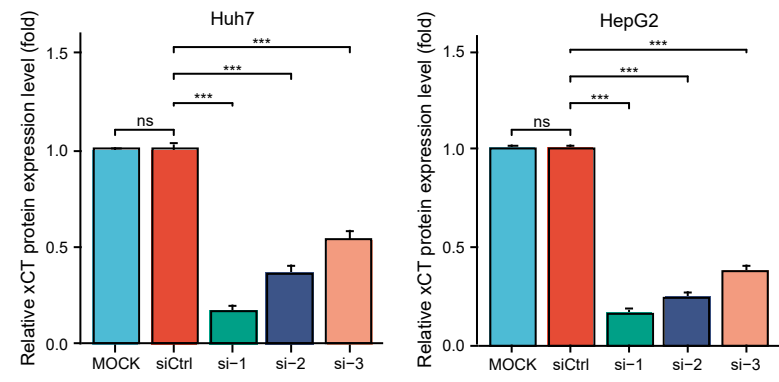

D

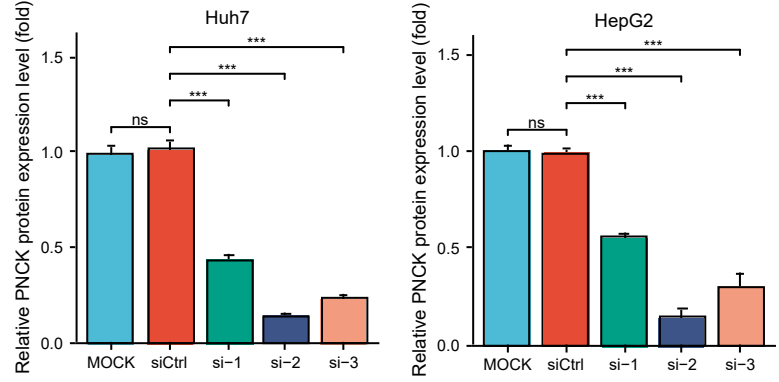

Supplement: Supplementary file 5 — Figure S5. [file CAM4-12-8526-s002.pdf]

Figure S6

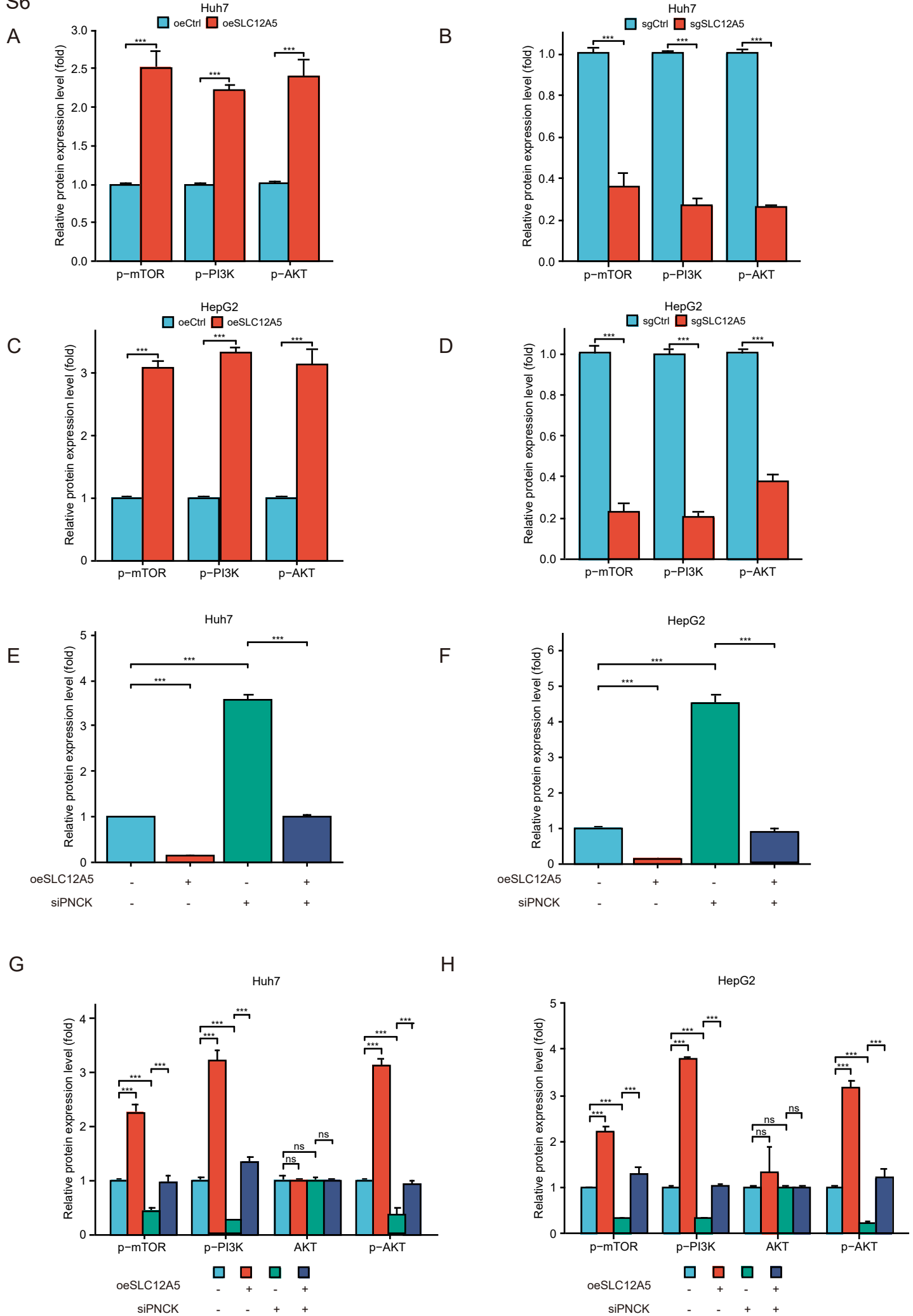

Supplement: Supplementary file 6 — Figure S6. [file CAM4-12-8526-s011.pdf]

Figure S7

A

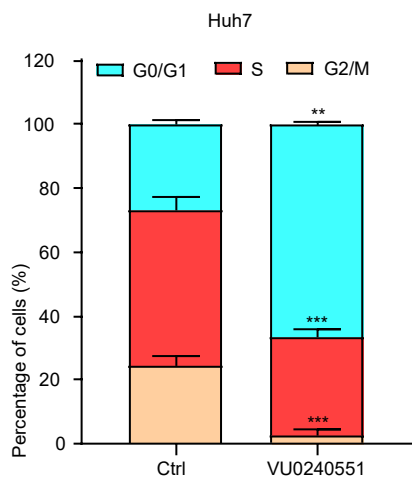

B

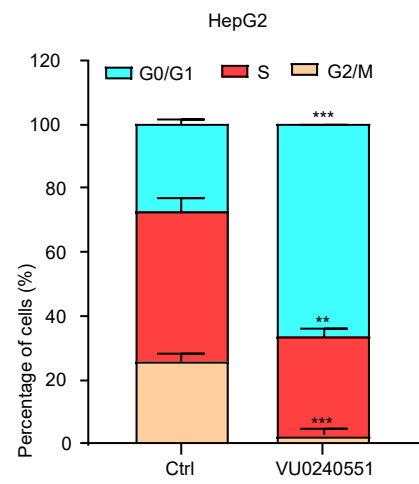

C

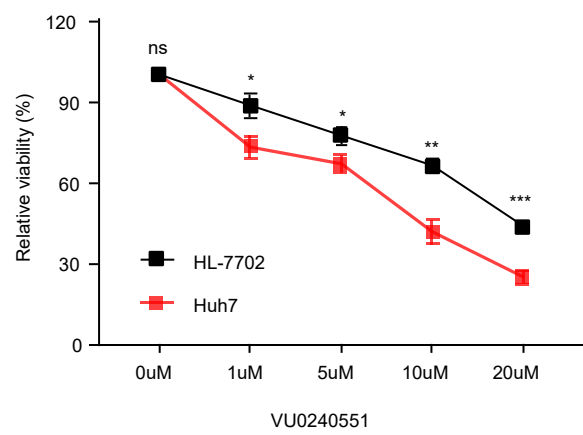

D

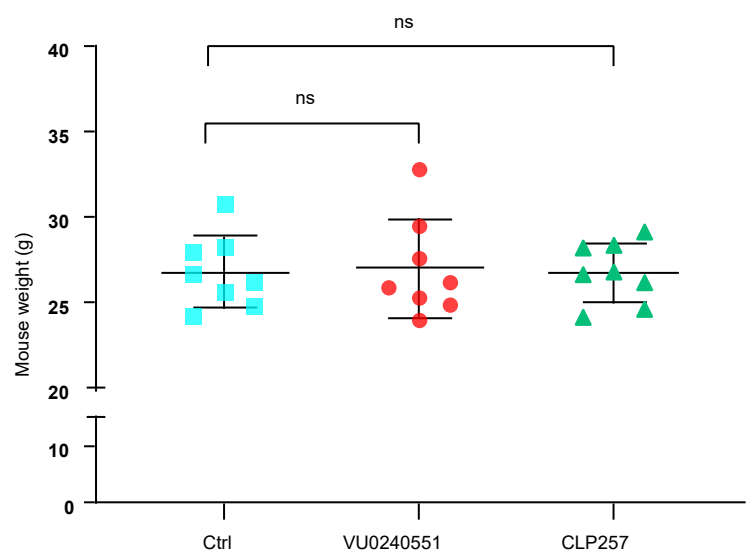

Supplement: Supplementary file 7 — Figure S7. [file CAM4-12-8526-s006.pdf]
